# Supplementary figures and images for: Lack of Endothelial α1AMPK Reverses the Vascular Protective Effects of Exercise by Causing eNOS Uncoupling
Source: Antioxidants (Basel). 2021 Dec 10;10(12):1974. doi: 10.3390/antiox10121974 (PMC8750041; doi:10.3390/antiox10121974)

## Supplemental Figure S1

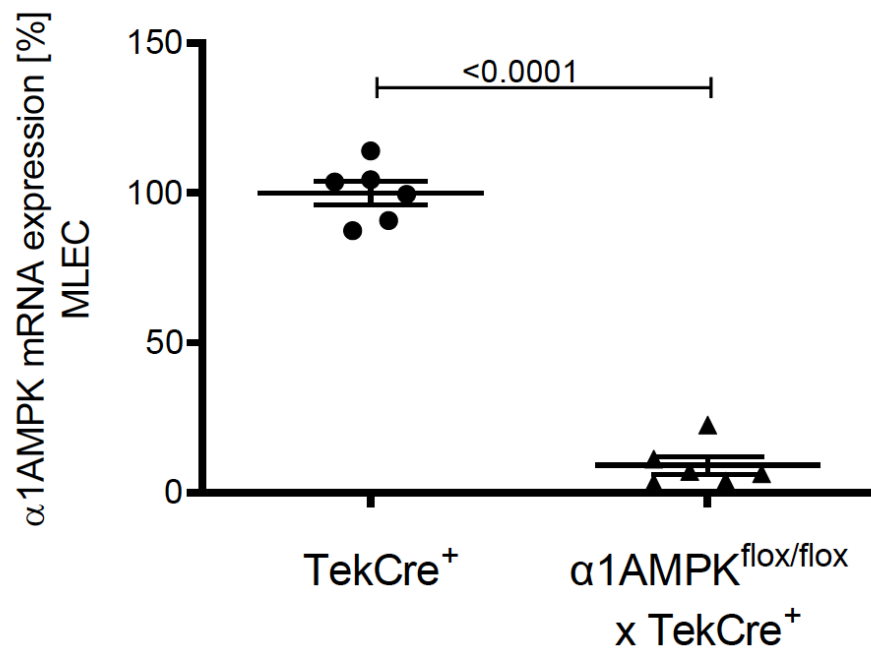

Figure S1. mRNA expression of  $\alpha 1$ AMPK in MLEC.

Supplement: Supplementary file 1 [file antioxidants-10-01974-s001.zip › antioxidants-1472923-supplementary.pdf]
